# Obtaining and conductive properties of a vanadate-borate-phosphate glass

**DOI:** 10.1038/s41598-023-43302-8

**Published:** 2023-09-25

**Authors:** Mihai Eftimie, Ana Violeta Filip, Cristian Beniamim Danescu, Andrei Nitescu, Bogdan Alexandru Sava

**Affiliations:** 1 Department of Science and Engineering of Oxide Materials and Nanomaterials, National University of Science and Technology “Politehnica” of Bucharest, 1 Polizu Street, District 6, 011061 Bucharest, Romania; 2 Lasers Department, National Institute for Laser, Plasma and Radiation Physics, 409 Atomistilor Street, 077125 Magurele, Ilfov County Romania; 3 National Institute of Materials Physics, 405 A Atomistilor Street, Magurele, Ilfov County Romania

**Keywords:** Glasses, Electrical and electronic engineering, Thermoelectric devices and materials

## Abstract

Vanadate glasses exhibit semiconducting property at certain temperatures. This work demonstrates the conductivity of the composition 45V_2_O_5_–25B_2_O_3_–30P_2_O_5_, which is a new glass in the vanadium-boron-phosphorus ternary system that expands the glass forming area reported in literature data. The glass was obtained through a classical melt-quenching technique. The structural composition of the obtained glass was revealed with Raman spectroscopy and the amorphous characteristic has been highlighted with X-ray diffraction. The characteristic temperatures and the thermal expansion coefficient were determined by dilatometry. Based on the experimental measurements of electrical resistance, mathematical calculations were performed, resulting in a conductivity of 2.04·10^−6^ S/cm at 125 °C, and an activation energy of 42.91 kJ/mol for this glass. Impedance spectroscopy in DC and AC at 100 V and 100 Hz to 2 MHz, respectively, showed a lower activation energy of about 0.166 eV and transition temperatures of 24 °C and 11 °C, respectively. These results were compared with those from the literature considering the temperatures at which the reported conductivities were measured. This glass has potential applications in electronic devices and temperature sensors.

## Introduction

Phosphorus oxide (P_2_O_5_) and boron oxide (B_2_O_3_) have recently attracted increasing interest as excellent glass formers with their low melting temperatures. Boro-phosphate glasses are characterized by improved chemical stability as well as high mechanical and optical properties^1–7^.

Conductive and semiconductive glasses have gained interest in the field of solid-state chemistry and materials science due to their potential application in power sources, photonics, gas sensors etc. These glasses usually contain transition metal oxides that possess electrical conductivity owed to polyvalent transition metal ions^8–13^. Vanadate glasses present the highest electron conductivity among oxide glasses, making them attractive for optoelectronic materials^1,4,8,14–18^. Vanadium oxide added in small amounts to phosphate glasses acts as a network modifier by depolymerizing long phosphate chains^2^. At high contents, V_2_O_5_ becomes a glass network former^3,19^. Rammah et al.^3^ obtained glasses in the V_2_O_5_–P_2_O_5_–B_2_O_3_ system with vanadium in molar percentages of 46–50% with an equimolar ratio V_2_O_5_:P_2_O_5_ of 1:1. Studies have also been conducted on the change in properties following the substitution of phosphorus oxide with boron oxide. Han et al.^19^ prepared glasses with 50 mol% of vanadium oxide while changing the molar percentage of phosphorus oxide from 50 to 10% by adding boron oxide. They showed how the variation of V^4+^ and V^5+^ species influences the conductivity of these glasses^19^. In another study, 50 mol% and 70 mol% vanadium oxide glasses were obtained^20^. Furthermore, a glass forming zone (vitrification zone) in the V_2_O_5_–B_2_O_3_–P_2_O_5_ ternary system was defined by Han^19^ and Choi^20^. Regarding the conductivity of vanadium glasses in the vanadate-borate-phosphate system, the authors, Choi et al.^20^ have revealed the electrical character of their samples by Hall effect measurements. The 60 mol% vanadium oxide glasses obtained by Barde et al. indicate an electrical conductivity that increases with temperature and with a molar percentage of boron oxide from 5 to 35% to the detriment of phosphorus oxide^10^.

This paper presents the study of the conducting properties of a vanadate-borate-phosphate glass with the formula 45V_2_O_5_–25B_2_O_3_–30P_2_O_5_ (mol%). This composition is outside the vitrification zone reported by Han^19^, Choi^20^ and Choi^21^. Thus, our work presents a new glass (evidenced as non-crystalline by XRD analysis) that manifests conductive properties at low temperatures, comparable to the reported ones in^8,14,16^ and extends the knowledge regarding the area of vitrification/glass forming that is reported in the literature^20,21^ for this ternary system. Furthermore, the glass was obtained from oxides rather than phosphoric acid or ammonium dihydrogen phosphate, as in other studies^20,22–25^. Dilatometry analysis was performed, and the electrical properties were measured and calculated. This glass has potential applications in electronic devices and temperature sensors.

## Material and methods

The precursors used, vanadium oxide (V_2_O_5_), boron oxide (B_2_O_3_), and phosphorus oxide (P_2_O_5_) were all of analytical grade, from Sigma-Aldrich. The 45V_2_O_5_–25B_2_O_3_–30P_2_O_5_ (mol%) glass was obtained through the melt-quenching method.

The precursors were weighed on an analytical balance, homogenized in an agate mortar and then transferred to a 100 ml sintered alumina crucible. The crucible was introduced into a MoSi_2_ resistive elements-equipped electrical oven.

**Figure 1:**
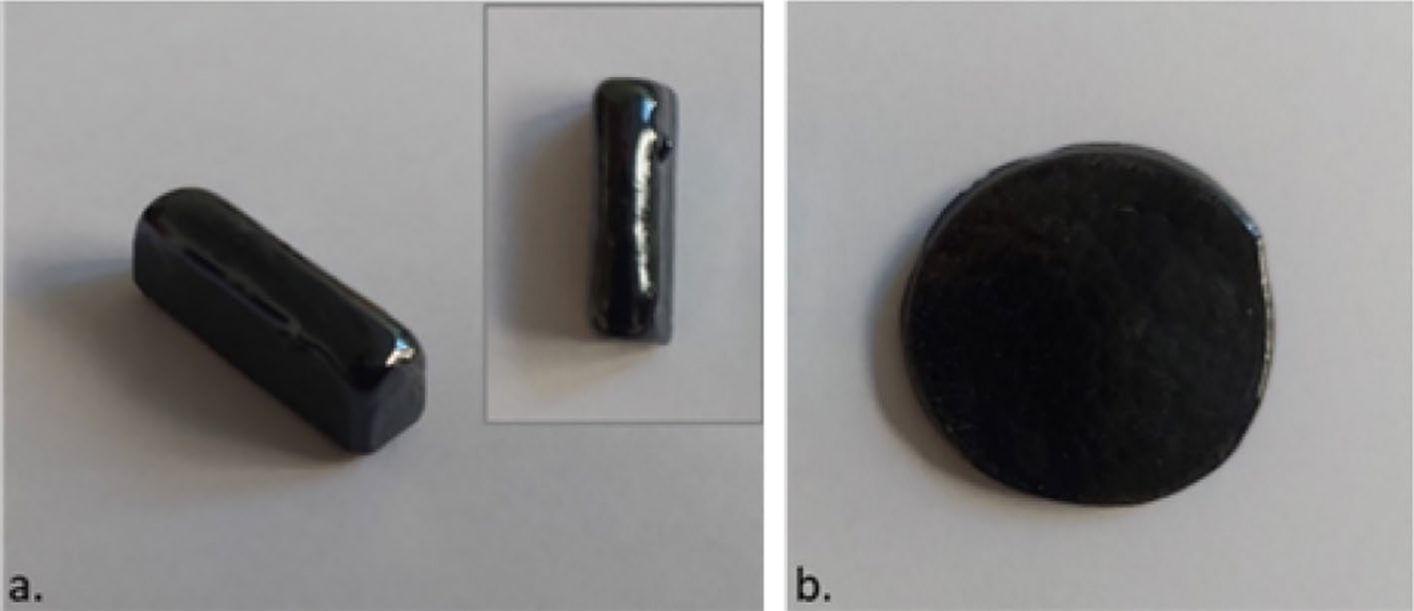
The obtained samples after glass casting in (**a**) prismatic and (**b**) disc shapes.

When the mixture reached the melting temperature of 950 °C, a plateau was maintained for 1 hour. The melt was then cast into a prismatic shape (for dilatometry analysis) and a disc shape for XRD and resistivity measurements (Fig. 1). The next step consisted of annealing the glass at 300 °C for 1h to reduce the thermal stresses of the glass. The resulting glass was cut, ground and polished to provide samples for analysis.

The sample was investigated by X-ray diffraction to demonstrate its amorphous characteristics. The diffractometer was an Empyrean diffractometer from Panalytical (Malvern, UK) operating with a generator power of 45 kV and 40 mA in a parallel beam geometry through a parabolic X-ray mirror for Cu Kα radiation and a 1/8° slit in the incident beam side and a parallel beam collimator of 0.27° in front of an X’celerator detector in the diffracted beam side. For Raman measurements, a LabRam HR Evolution HORIBA (Palaiseau, France) spectrometer was used. The Raman spectrometer has a 514 nm laser, and the acquisition time was 2 seconds. The hole diameter is 100 µm, the objective 50×, the grating of 600 gr/mm, and the range is between 100–16,000 cm^−1^, with a measurement error of ±0.5 cm^−1^. The characteristic temperatures and thermal expansion coefficient (${\alpha }_{20}^{200}$) of the obtained glass were determined using a horizontal Netzsch DIL 400 PC dilatometer, NETZSCH Holding, Selb, Germany, equipped with a Proteus software for characteristic temperatures and thermal expansion coefficient calculation. The resistivity was measured using a Fluke 115 True RMS Multimeter with an accuracy of ±0.9%, coupled to a small furnace equipped with a thermocouple and two 1.6 cm diameter electrodes that are in contact with the two opposite faces of the sample, also 1.6 cm in diameter. The dielectric spectroscopy measurements were carried in vacuum, at temperatures between 50 and 495 K (− 223 °C, 221.8 °C), with a 1V amplitude a.c. signal by using a HIOKI IM3536 impedance analyzer (Z ±0.05% rdg. θ: ±0.03°). The sample was heated up to 293 K afterwards performing measurements during cooling down to 50K, with a constant cooling rate of 1K/min. The direct current measurements were carried in vacuum, at temperatures between 30 and 480 K (− 243 °C, 206.8 °C) using a Keithley 6517 (10*10^−18^ A current measurement resolution). The sample was cooled down to 30K under an applied bias of − 100 V afterwards measuring during heating up to 480K with a constant heating rate of 1 K/min.

## Results and discussion

**Figure 2:**
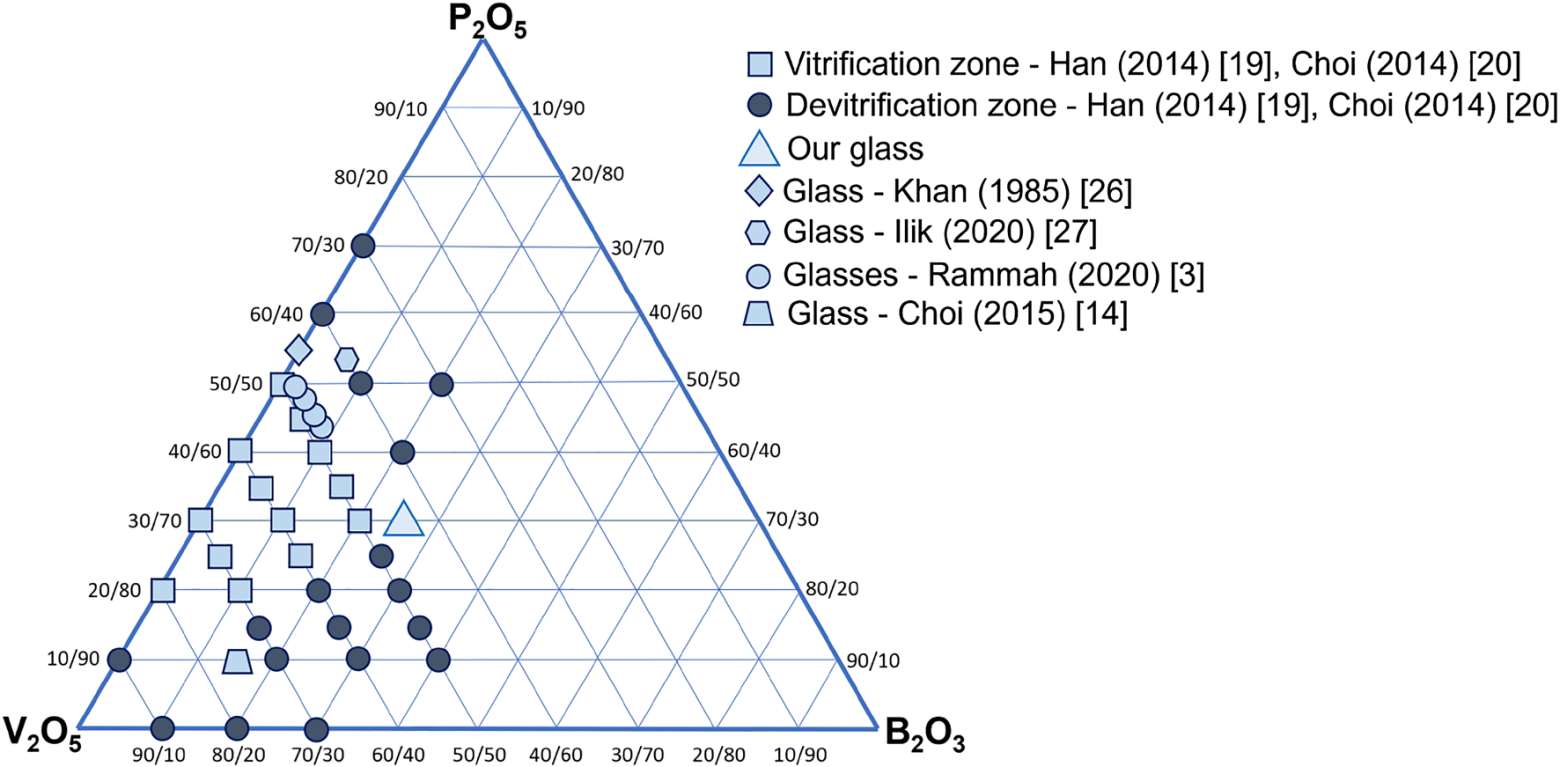
Diagram for V_2_O_5_–B_2_O_3_–P_2_O_5_ system ^3,14,19,20,26,30,32^.

Figure 2 presents the ternary diagram of glasses in the V_2_O_5_–B_2_O_3_–P_2_O_5_ system, along with our composition (marked by a triangle) that extends the vitrification zone, as denoted by other researchers in the literature.

### The X-Ray diffraction

**Figure 3:**
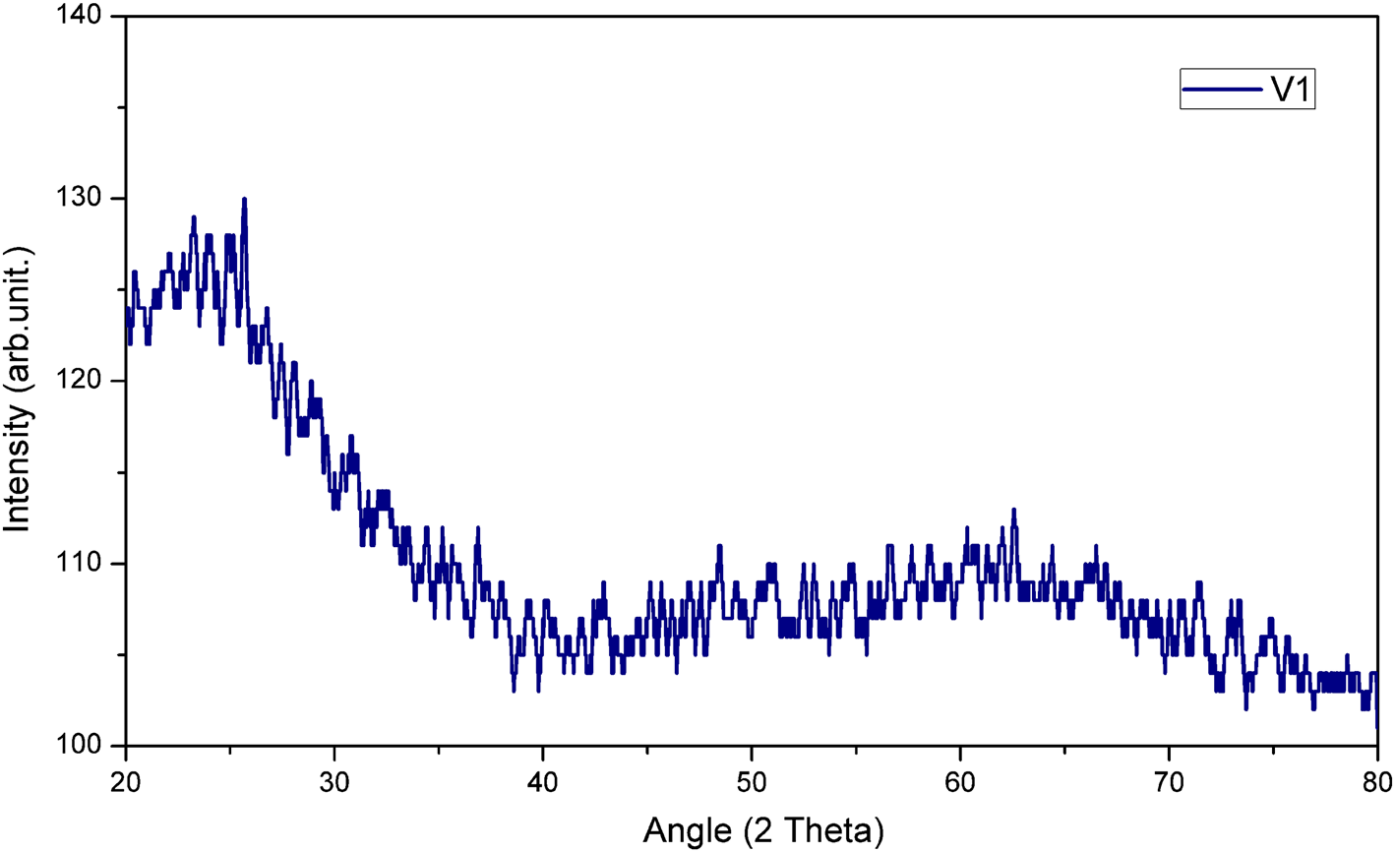
The XRD diffractogram for 45V_2_O_5_–25B_2_O_3_–30P_2_O_5_ glass.

Figure 3 shows the X-ray diffraction (XRD) pattern of the 45V_2_O_5_–25B_2_O_3_–30P_2_O_5_ glass (the sample from Fig. 1b). As the pattern contains no sharp peaks, we concluded that a non-crystalline material was obtained, as intended and that no free crystalline oxides are present in the system. Their presence, if signaled by specific peaks, would have required the reprocessing of the sample. Therefore, the 45V_2_O_5_–25B_2_O_3_–30P_2_O_5_ glass was correctly prepared and annealed and ready to be used for the analyses that investigate the structure and properties of interest.

### The dilatometry analysis

This analysis method is the best method for determining the behavior of glass against thermal stresses. Thermal expansion is characterized by the linear expansion coefficient that depends on the composition of the glass (the energy of the chemical bonds) and directly influences the thermal shock resistance. The expansion coefficient depends on the sample shape (that must have parallel plane faces) and the thickness of the glass sample. That is the reason the analysis was carried out on the sample presented in Fig. 1a. This method also determines the specific temperatures of a vitreous material: strain temperature (T_IR_); glass transition temperature (T_g_) (above this temperature the viscosity of glass decreases, and atoms tend to rearrange); annealing temperature (T_SR_); dilatometric softening temperature (T_D_). These temperatures are determined by the dilatometer software from the plot of sample elongation (dL/L_0_) versus temperature (where L_0_ is the initial sample length) graph^18,26^.

**Figure 4:**
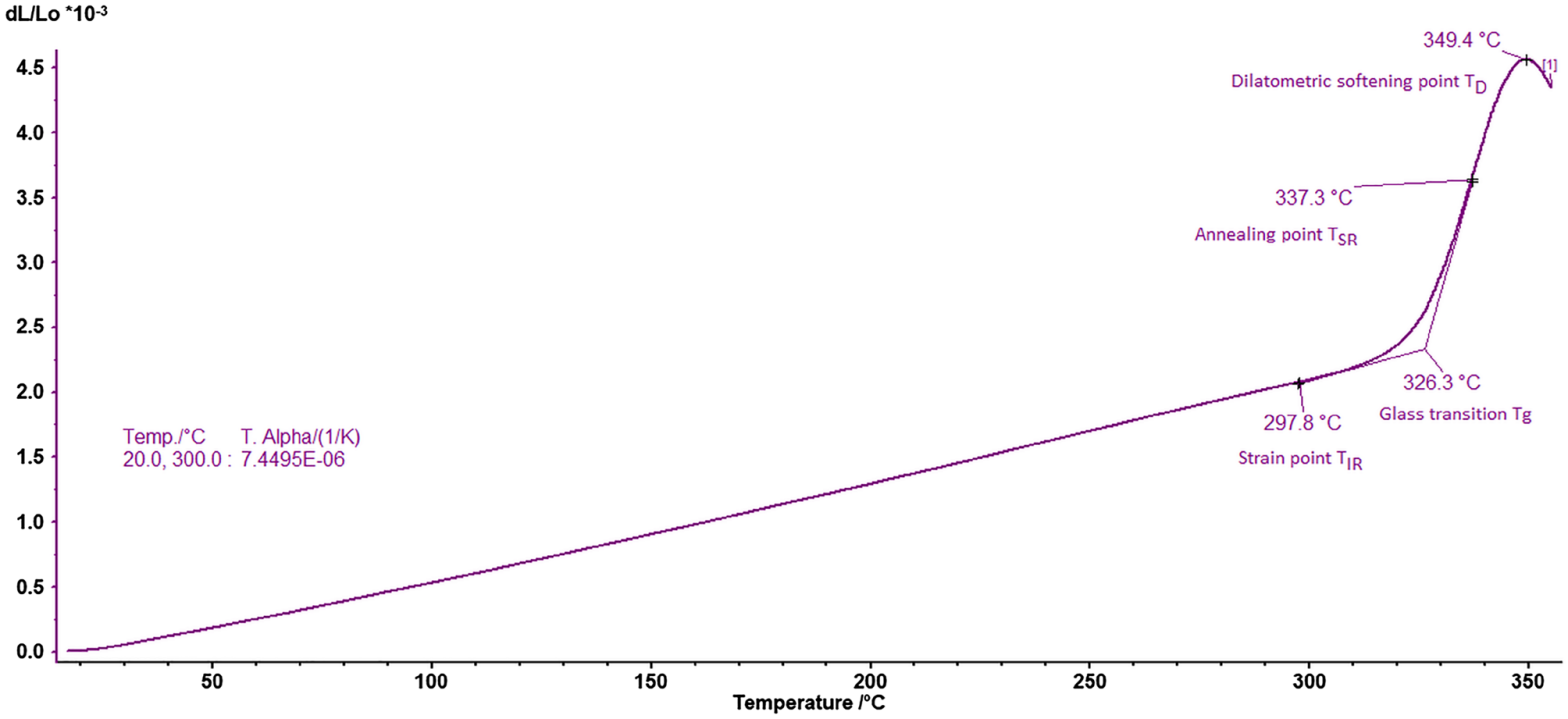
The 45V_2_O_5_–25B_2_O_3_–30P_2_O_5_ glass dilatometry graph containing the characteristic temperatures, strain temperature (T_IR_); glass transition temperature (Tg); annealing temperature (T_SR_); dilatometric softening temperature (T_D_) and the expansion coefficient T. Alpha (1/K).

The thermal expansion graph for the 45V_2_O_5_–25B_2_O_3_–30P_2_O_5_ glass is represented in Fig. 4, together with the specific temperatures, as indicated by the Proteus software of the dilatometer, and the linear expansion coefficient. From the inflection of the curve, the value of glass transition temperature (T_g_) is obtained. Up to this value, the glass behaves like vitreous solid, while above this temperature the structural mobility of the glass is high enough to allow structural transformations. Therefore, T_g_ is an indicator of the structural stability of vitreous state. The higher the T_g_, the greater the range of temperatures where the glass can be used, whilst a glass with lower T_g_ and conductivity at low temperatures can be used in temperature sensors^18,26^.

The T_g_ of the 45V_2_O_5_–25B_2_O_3_–30P_2_O_5_ glass is 326.3 °C (Fig. 4), much lower than the Tg for usual glasses (for example the usual soda-lime glass has Tg between 520 and 600 °C) and also lower than the T_g_ of other vanadium containing glasses, such as natrium-titanium-vanadium phosphate glass, 289–432 °C^27^, vanadium-boro-tellurite glass, 329–354 °C^28^, vanadium-lithium-borate glass, 345–419 °C^29^, bismuth-phospho-borate-vanadate glass, 350-490 °C^2^, silver-doped vanadium boro-phosphate glass, 420–431 °C^30^, vanadium-zinc-phosphate glass, 426–463 °C^31^, RE-doped doped vanadium phosphate glass, 300-500 °C^32^ or vanadium-phosphate glass, 365–557 °C^8^. This lower value of T_g_ can signify a lower activation energy for the conductive properties, as seen for two of the above literature glasses, RE-doped doped vanadium phosphate glass, 0.45–0.57 eV^32^ and vanadium-phosphate glass, 48.7 kJ/mol^8^.

The annealing temperature must be between T_IR_ and T_SR_ to remove the stresses in the glass^18,33^. The 45V_2_O_5_–25B_2_O_3_–30P_2_O_5_ glass was annealed at 300 °C, which is between the strain and annealing temperatures (T_IR_ and T_SR_—according to Fig. 4), which is the temperature range for annealing as presented by Balta^18^. Prolonged annealing increases the density and, in some cases, the electrical conductivity of glasses^34,35^. The sample didn't show any cracks, which means that the annealing reduced the internal thermal stresses enough for further processing.

### Raman analysis

**Figure 5:**
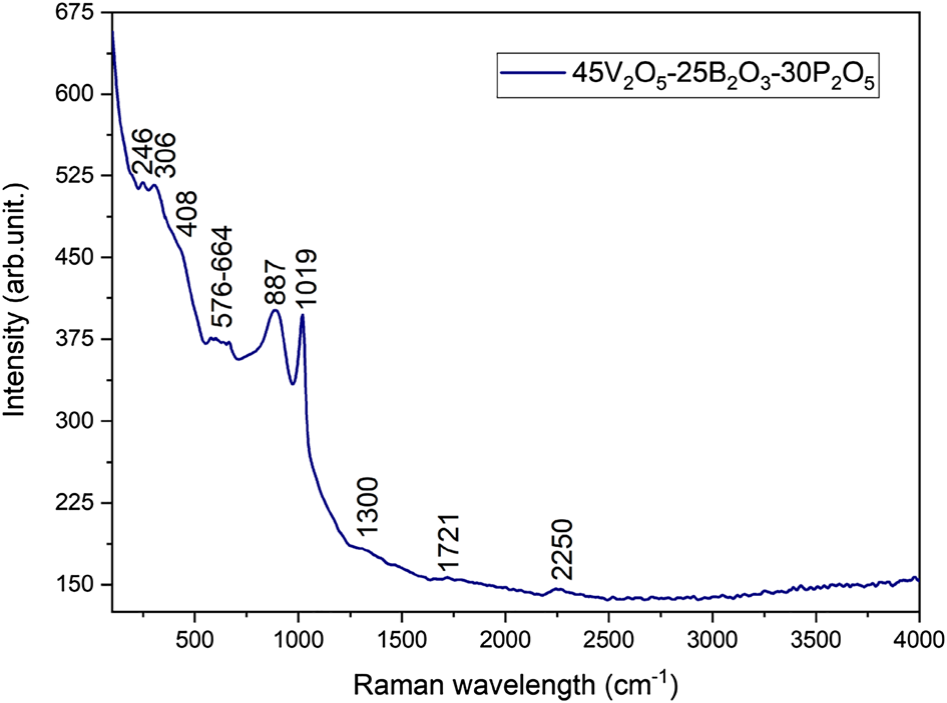
The Raman spectra for 45V_2_O_5_–25B_2_O_3_–30P_2_O_5_ glass.

The Raman spectra was obtained at room temperature, in the range from 200 to 4,000 cm^-1^ and is presented in Fig. 5.

The peaks from 246, 306, and 408 cm^−1^ correspond to O–P–O bending vibrations in PO_2_ groups^36,37^. The band at 246 cm^−1^ can also be assigned to the bending vibrations of O–VO_3_^38^. The Raman band at 300 cm^−1^ is attributed to the bending vibrations of V–O–V bonds^39^. The low intensity bands between 576 and 664 cm^−1^ (the peaks are: 576 cm^−1^, 601 cm^−1^, 632 cm^−1^, and 664 cm^−1^) are assigned as follows: at 576 and 601 cm^-1^ the bending modes of the orthophosphate PO_4_^3−^ unit (O–P–O vibrations) are found^36,40^; at 632 cm^−1^ has been ascribed to the vibrations of P–O–B^2^ and V–O–P^31^ bridges; the band from 664 cm^−1^ shows the presence of B–O–B units^25^ and V–O stretching vibration^28,39^.

The bands around 880 cm^−1^ correspond to the vibrations of B–O in B(OH)_3_, to the stretching vibrations of P–O–P bonds, and to the pyroborate groups ^36^. The band at 887 cm^-1^ is attributed to the V–O stretching vibration modes or to BO_4_ unit^2,28,41^. Hejda and co-authors demonstrated in their paper^31^ that the peaks from 800 to 1100 cm^−1^ are especially due to the V_2_O_5_ content that creates the glass lattice because these peaks occur only when vanadium becomes the network former, a statement supported by other studies^15,27,31^. The bands between 800 and 1100 cm^−1^ can be also attributed to the symmetrical and asymmetrical vibrations of the PO_4_ and VO_4_ groups^27^. The Raman sharp band at about 1020 cm^−1^ is attributed to V^5+^=O stretching vibration of tetragonal pyramid VO_5_^39,41^ The band can be attributed also to stretching vibrations of BO_4_, to the pyrophosphate units due to P_2_O_7_^4−^ ions and orthophosphate units^27,36,42^.

The P=O stretching mode band is assigned at 1317 cm^−1^, but due to the boron oxide presence, the band shifts to 1300 cm^−1^^25,36^. At 1300 cm^−1^ and 1721 cm^−1^ there are vibrations belonging to the boron structure: BO_3_ unit, B–O–B stretching, and B–O^−^ stretching vibration between non-bridging oxygen and B in the BO_3_ structure^28,42^. The low intensity band at 2250 cm^-1^ is attributed to the bending vibrations of residual hydroxyl groups in B(OH)_3_^43^.

### Electrical resistivity and conductivity—direct measurements

The glass (from Fig. 1b) was mechanically processed to produce a sample 1.6 cm in diameter and 3 mm thick, with polished parallel planar surfaces for the best contact between the sample and the electrodes of the apparatus used. In our experiments, the electrodes of the apparatus used had the same diameter as the sample, 1.6 cm. During the experiment, the dependence of the electrical resistance of the sample (Re) as a function of temperature is measured using direct current (DC).

The volume electrical resistivity (ρ) is deduced from the relation^18^:

1 $$\rho =Re\times \left(S/l\right)$$

where Re is the resistance in volume of the glass measured with the multimeter, S is the surface area of the sample and *l* is the thickness of the glass sample.

The volume electrical conductivity (σ) of glass is the reciprocal of the volume resistivity^18^:

2 $$\sigma =\frac{1}{\uprho }=\left(1/Re\right)\times \left(l/S\right)$$

The electrical resistance was measured in DC at 0.2 V, at different temperatures, resulting the data in Table 1, together with the calculated resistivity, conductivity, log σ and 10^3^/T.

**Table 1:** The results of the calculated electrical conductivity (σ).

T °C	T °K	10^3^/T K^-1^	Re Ω	ρ Ω cm	σ S/cm	log σ log (S/cm)
125	398	2.51	73000	489250.7	2.04 × 10^−6^	− 5.690
130	403	2.48	60300	404134.5	2.47 × 10^−6^	− 5.607
135	408	2.45	50900	341135.1	2.93 × 10^−6^	− 5.533
140	413	2.42	42500	284837.7	3.51 × 10^−6^	− 5.455
145	418	2.39	38500	258029.5	3.88 × 10^−6^	− 5.411
150	423	2.36	33400	223848.9	4.47 × 10^−6^	− 5.350
155	428	2.33	29400	197040.7	5.08 × 10^−6^	− 5.294
160	433	2.31	25300	169562.2	5.9 × 10^−6^	− 5.229
165	438	2.28	22200	148785.8	6.72 × 10^−6^	− 5.173
170	443	2.25	19300	129349.8	7.73 × 10^−6^	− 5.112
175	448	2.23	16900	113264.9	8.83 × 10^−6^	− 5.054
180	453	2.21	14000	93828.9	1.067 × 10^−5^	− 4.972
185	458	2.18	12300	82435.39	1.213 × 10^−5^	− 4.916
190	463	2.15	12000	80424.77	1.243 × 10^−5^	− 4.906

**Figure 6:**
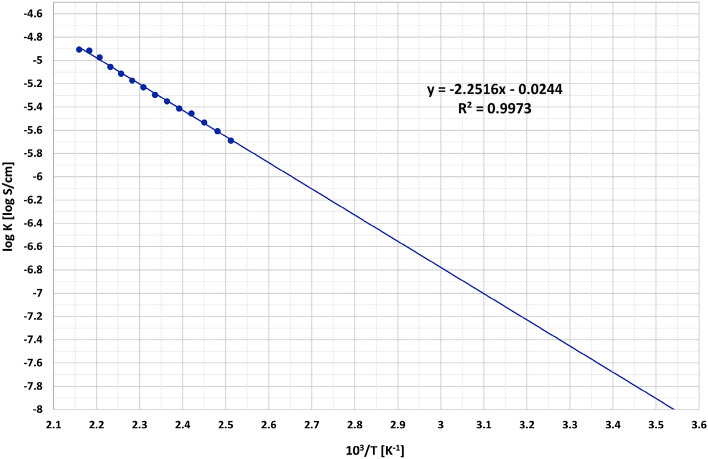
Logarithmic conductivity versus 10^3^/T for the 45V_2_O_5_–25B_2_O_3_–30P_2_O_5_ glass.

To determine the temperature at which the glass becomes semiconductor and to calculate the activation energy, the Arrhenius plot of the logarithm of the conductivity as a function of 10^3^/T is presented in Fig. 6, using the data from Table 1. From the graph in Fig. 6, the slope of the graph, m, is obtained from the equation of the regression line.

The calculus of the activation energy of the electrical conduction is carried out as the logarithm of equation^4,10^:

3 $$\sigma =A {e}^{-\frac{{E}_{c}}{RT}}$$

that leads to the next equation^12,18^:

4 $$\mathrm{log\sigma }=\mathrm{logA}-\left({E}_{c}/RT\right)\times \mathrm{loge}=\mathrm{logA}-\left[\left(0.43429\times {E}_{c}\right)/\left({10}^{3}\times R\right)\times \left({10}^{3}/T\right)\right]$$

So, the slope of the plot, m, is^26^:

5 $$\mathrm{m}=-\left(0.43429\times {E}_{c}\right)/\left({10}^{3}\times R\right)$$

where R is the universal gas constant (R=k·N, where k is Boltzmann’s constant of 1.3804×10^−23^ J/K and N is Avogadro's number of 6.023×10^23^ mol^−1^). From Eq. (5) it results^26^:

6 $${\mathrm{E}}_{\mathrm{c}}=\left({10}^{3}\times R\times m\right)/-0.43429$$

Ec is the activation energy for electrical conductivity, expressed in J/mol. Considering the articles of other authors^4,10,44–47^, some vanadate and phosphate-tellurite glasses can exhibit also ionic conductivity in addition to the much more important electronic conductivity. This ionic conductivity is due to the migration of non-bridging oxygen along the network-former chains^45,46^ or to the ion hopping of oxygen vacancies^47^. In their rigid state, at room temperature, common glasses, such as flat glass or glassware, have a conductivity of about 10^-11^ S/cm, which puts them in the category of insulators, but there are also semiconducting glasses with a conductivity of up to 10^−5^ S/cm^48^. To compare the glasses in terms of electrical conductivity, the temperature at which the conductivity value is 10^-8^ S/cm (10^-6^ S/m) can be used as an indicator of the conductivity limit between dielectrics and semiconductors [18, https://spark.iop.org/conductivity-electrical].

The electrical conductivity values of 45V_2_O_5_–25B_2_O_3_–30P_2_O_5_ glass were determined experimentally and are presented in Table 1 up to the maximum temperature of 190 °C permitted by the experimental setup. At 150 °C the conductivity of obtained glass is 4.47·10^−6^ S/cm, with is comparable to Saiko et al.^8^ results: at 150 °C the conductivity is 1.89·10^−6^ ± 8.37·10^−8^ S/cm for sample 45V_2_O_3_–55P_2_O_5_. Saetova et al.^16^ obtained similar results for 0.3Li_2_O − (0,7 − x)B_2_O_3_ − xV_2_O_5_, with x = 0.45, namely a conductivity between 3.3·10^−5^ ± 3.6·10^−8^ S/cm at 170 °C.

According to Table 1 data, the obtained glass has a resistance of only 73·10^3^ Ω at 125 °C and became semiconductor at a lower temperature, since at 125 °C the conductivity value is 2.04 · 10^-6^ S/cm, much higher than the considered limit value of 10^-8^ S/cm.

From the extrapolation of the graph (Fig. 6) the value for 10^3^/T and, implicitly, for T, corresponding to log_σ_ = − 8 (for 10^−8^ S/cm), is obtained. This gives a value for 10^3^/T of 3.54 and, consequently, a temperature of 9 °C at which the glass becomes a semiconductor , meaning that the developed glass has electrical properties suitable for the temperature sensing field at room temperature. The activation energy value (E_c_) of the glass is calculated using Equation 6 and has a value of 42.91 kJ/mol, which corresponds to an activation energy of 0.44 eV, making this glass suitable for use in photoelectronic applications. This result is comparable to the work of Khan^32^, Saiko^8^ and Saetova^16^ namely: for samples with 45 % V_2_O_5_ the Ec values are 0.45-0.57 eV^32^, for sample 45V_2_O_3_–55P_2_O_5_^8^ the E_c_ value is 48.7 kJ/mol; for sample 0.3Li_2_O–(0.7 − x)B_2_O_3_ − xV_2_O_5_^16^, where x is 0.45, the E_c_ value is 41.0 kJ/mol.

### Electrical properties from impedance measurements

From impedance spectroscopy measurements, the real and imaginary part of impedance and dielectric permittivity, together with the dielectric loss and conductivity, and with the activation energy E_c_, were measured and calculated for AC and DC, on a large scale of temperatures, between 50 to 495 K and 30 to 480 K, respectively. For AC the frequency was varied between 100 Hz and 2 MHz.

The equations used are well known and indicated in many papers^44,49^:

7 $$Z={Z}{\prime}+i*Z"$$

where Z = impedance, Z’ the real part and Z” the imaginary part of the impedance:

8 $${Z}{\prime}=Z*\mathrm{cos}\theta$$

9 $$Z"=Z*\mathrm{sin}\theta$$

The dielectric permittivity *ε* is expressed:

10 $$\varepsilon ={\varepsilon }{\prime}-i\varepsilon "$$

here *ε’* is the real part and *ε”* is the imaginary part of the dielectric permittivity:

11 $${\varepsilon }{\prime}=\frac{C*l}{S*{\varepsilon }_{0}};\varepsilon "=\varepsilon {\prime}*\mathrm{tan}\delta$$

where: l = thickness of sample

S = area of sample

*ε*_*0*_ = permittivity of vacuum~8.85*10^−12^ F/m

C is the capacitance:

12 $$C=\frac{Z"}{{Z}^{2}*2\pi f}$$

with: f = frequency.

Dielectric loss, tan *δ,* are calculated as:

13 $$\mathrm{tan}\delta ={Z}{\prime}/Z"$$

**Figure 7:**
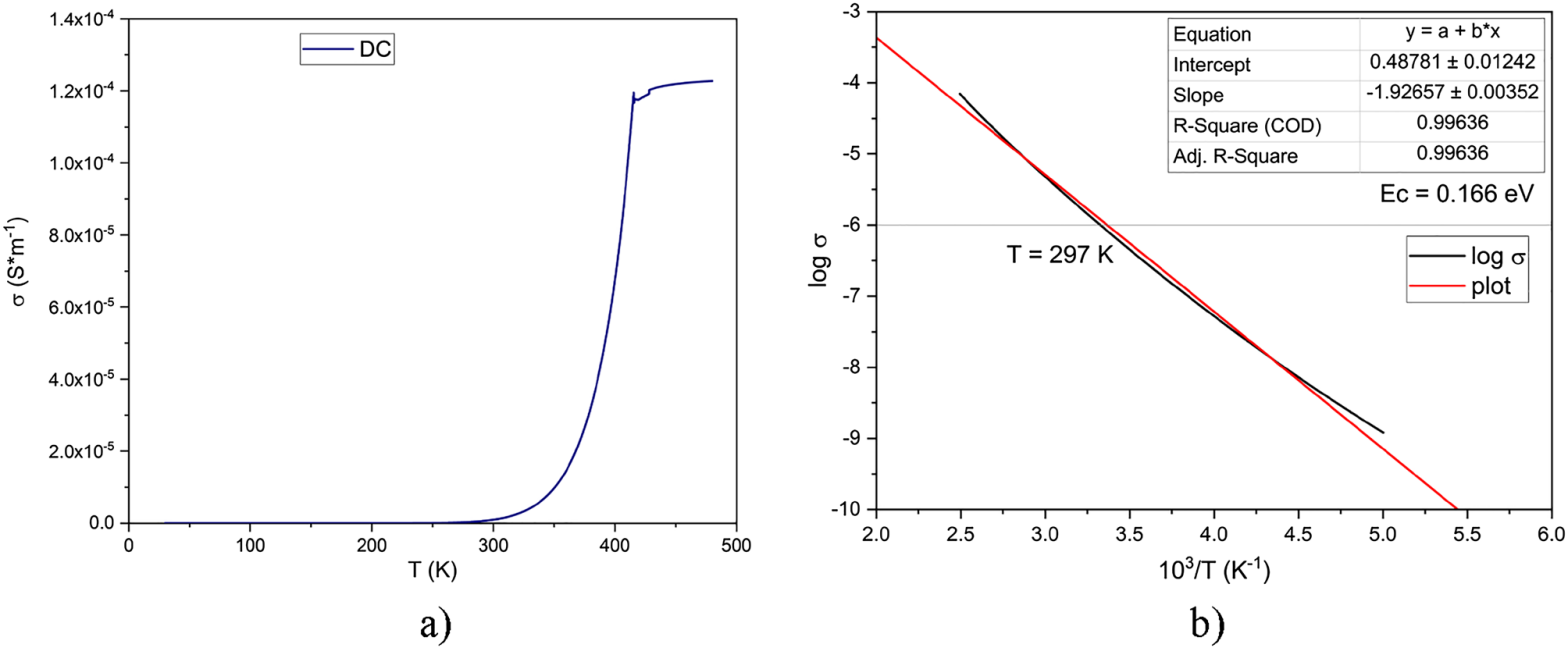
(**a**) The conductivity variation with temperature, in DC; (**b**) Arhhenius plot of logarithmic conductivity to 10^3^/T.

The variation of conductivity with temperature in the case of DC, 100 V, is shown in Fig. 7a. It can be seen that as the temperature increases, the conductivity increases for all temperature ranges up to 480 K.

Since the entire temperature range is below T_g_, the Arrhenius law of variation is applicable, and the Arrhenius plot is shown in Fig. 7b. From the slope of the curve as seen in Fig. 7b, we calculated the activation energy in a similar way as in the case of resistivity measurements below 1 V and obtained a similar value of 0.166 eV and a temperature for the dielectric-semiconductor transition of 24 °C.

**Figure 8:**
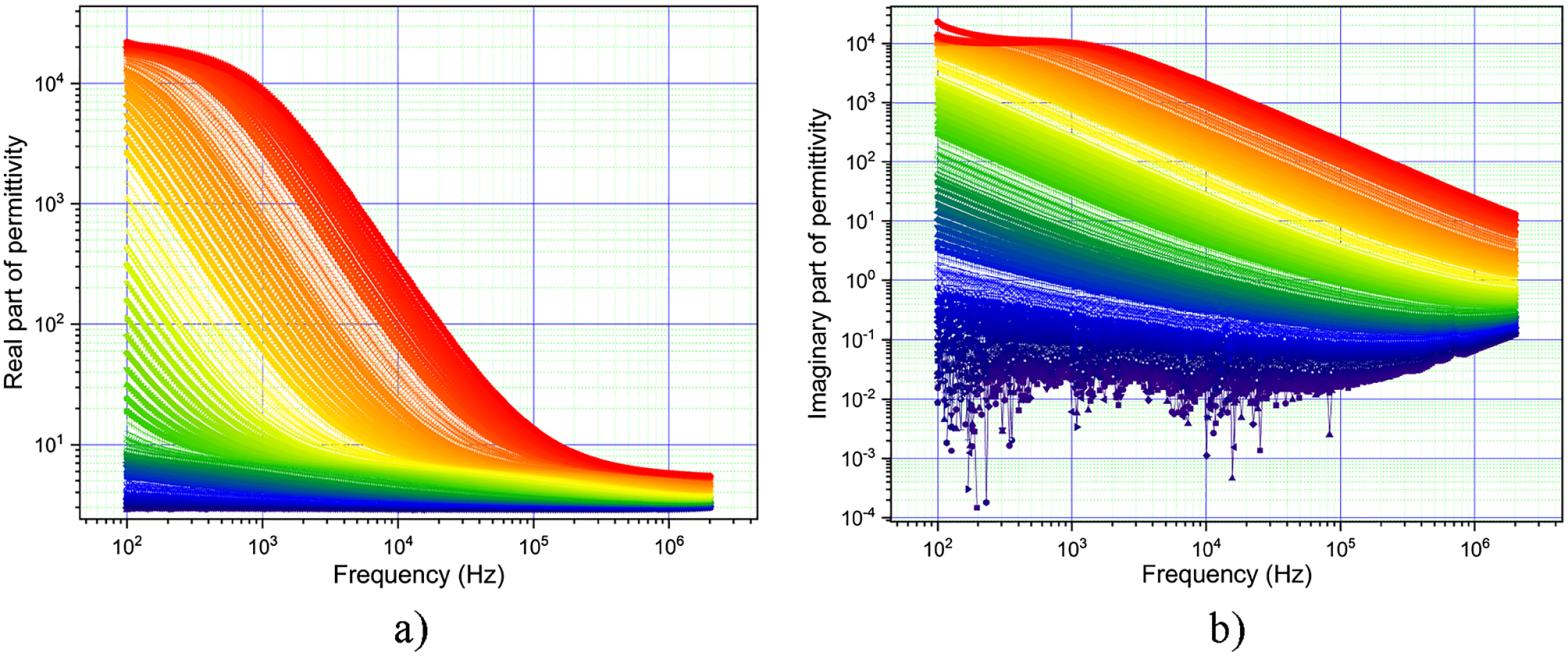
(**a**) The real part of dielectric permittivity variation with frequency and temperature; (**b**) The imaginary part of dielectric permittivity variation with frequency and temperature. The temperature increases from blue to red in figure.

For AC at frequencies between 100 Hz and 2 MHz, the variation of the real and imaginary part of the dielectric permittivity for the temperature range between 50 and 495 K as shown in Fig. 8.

As can be seen in Fig. 8, the real and imaginary parts of the permittivity decrease with frequency for all temperatures. This decrease become sharp for temperatures close to room temperature. It is also visible that both the real and imaginary parts of the dielectric permittivity increase with temperature for all domains.

**Figure 9:**
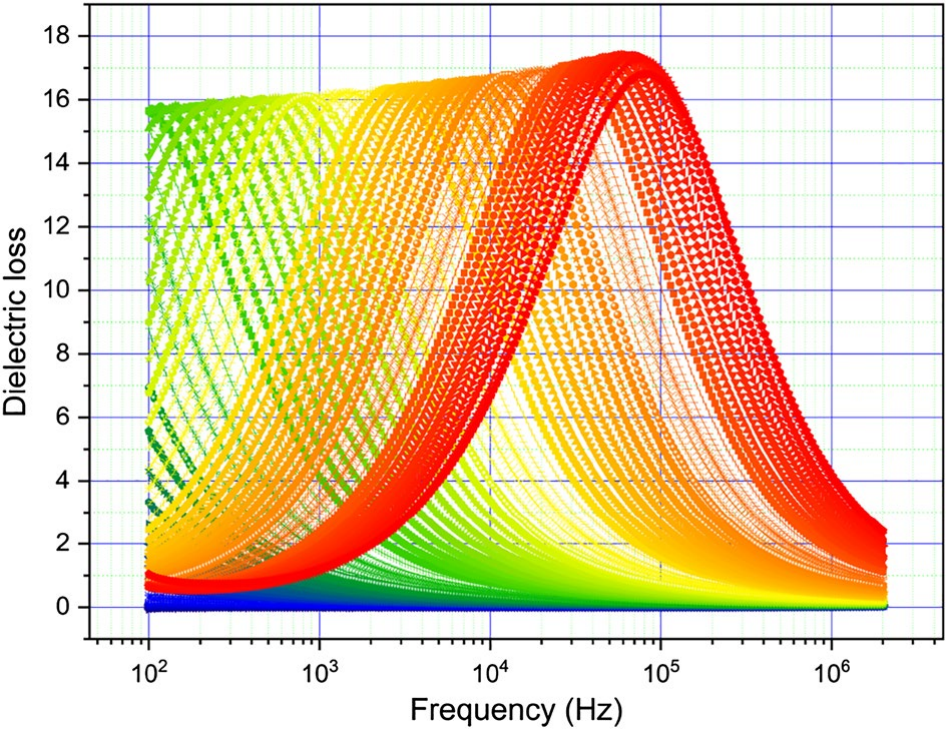
The dielectric loss versus frequency and temperature. The temperature increases from green to red.

The dielectric loss is shown in Fig. 9 for the same temperatures and frequency ranges.

**Figure 10:**
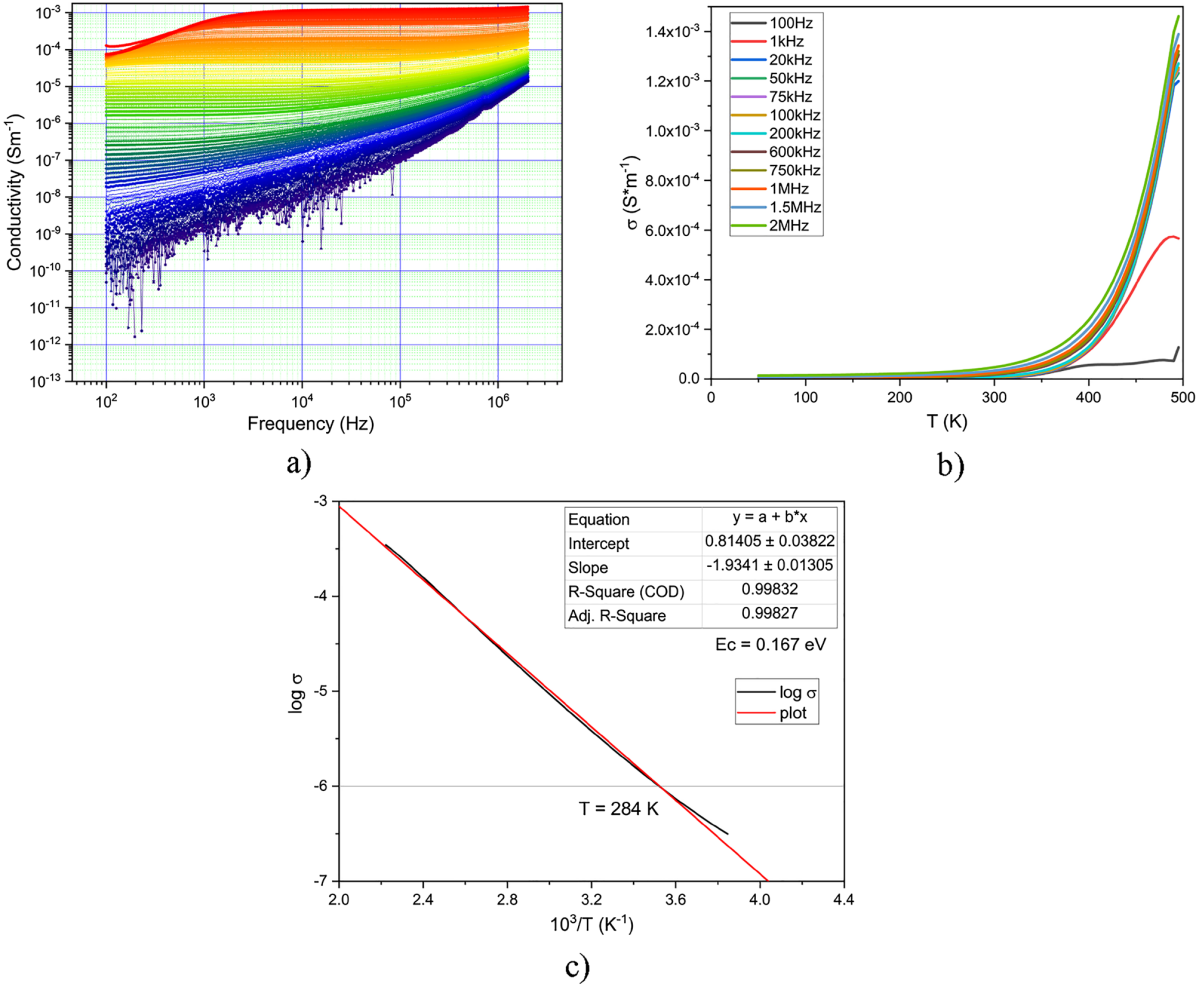
(**a**) Conductivity variation with frequency and temperature. Temperature increases from blue to red; (**b**) Conductivity variation with temperature at several frequencies; (**c**) Arrhenius plot of logarithmic conductivity with 10^3^/T.

The dielectric loss maximum increases slightly with frequency and moves to higher frequency with increasing temperature. This type of evolution of dielectric loss is close to that observed by Barde^4,10^. Conductivity versus frequency and temperature, together with the Arrhenius plot of logarithmic conductivity with 10^3^/T, are shown in Fig. 10.

AC conductivity increases with both temperature and frequency. The increase with frequency decreases and the variation becomes nearly linear at temperatures around 100 °C. The increase with temperature becomes sharp at temperatures around 100 °C and frequencies above 100 Hz. The increase of conductivity with temperature is signaled also by Barde^10^ for vanadium-boron-phosphate glasses with 60 % vanadium oxide. The linear variation of the conductivity with temperature can indicate the presence of an ionic charge transfer mechanism, due to the migration of non-bridging oxygen, via an activated hopping mechanism^44–47^.

From the slope of the Arrhenius plot shown in Fig. 10c, we calculated the activation energy in a similar way as in the case of resistivity measurements below 1 V and obtained a similar value of 0.167 eV and a temperature for the dielectric-semiconductor transition of 11 °C.

The transition temperature is close to that obtained for DC and to that obtained from resistivity measurements. The activation energy in both DC and AC impedance measurements is significantly lower than that obtained from resistivity measurements. This is probably due to the different voltages applied. This leads to the conclusion that the activation energy decreases with increasing voltage in both DC and AC for this glass sample.

## Conclusions

A new glass with the molar composition 45V_2_O_5_–25B_2_O_3_–30P_2_O_5_, that extends the vitrification area proposed by Han and Choi, was elaborated through the melt-quenching technique at a low melting temperature of 950 °C. The non-crystalline character of the samples was established by XRD analysis, while the accuracy of the annealing temperature (300 °C), known to influence the electrical conductivity, was confirmed through dilatometry.

Mathematical calculations based on DC and 0.2 V resistivity measurements were performed to emphasize the semiconducting nature of the developed glass. The resulting dielectric-semiconductor temperature is 9 °C, with an activation energy as low as 0.44 eV, making the glass usable in room temperature photoelectronic devices. Impedance spectroscopy in DC and AC at 100 V and 100 Hz to 2 MHz, respectively, showed a lower activation energy of about 0.166 eV and transition temperatures of 24 °C and 11 °C, respectively. In both DC and AC cases, the conductivity increases with temperature and frequency sharply at temperatures of above 100 °C. All these properties and technological parameters show that this glass is easier to obtain compared with other semiconductive glasses and demonstrate that it can be used in temperature sensors due to its semiconductive character at room temperatures.

## Data Availability

All data generated or analyzed during this study are included in this published article .
